# Immune competence and spleen size scale with colony status in the naked mole-rat

**DOI:** 10.1098/rsob.210292

**Published:** 2022-04-06

**Authors:** Valérie Bégay, Branko Cirovic, Alison J. Barker, Robert Klopfleisch, Daniel W. Hart, Nigel C. Bennett, Gary R. Lewin

**Affiliations:** ^1^ Max-Delbrück-Center for Molecular Medicine in the Helmholtz Association (MDC), Laboratory for Molecular Physiology of Somatic Sensation, Robert-Rössle Straße 10, D-13125 Berlin, Germany; ^2^ Division of Cellular Immunology, German Cancer Research Center, Im Neuenheimer Feld 280, 69120 Heidelberg, Germany; ^3^ Institute of Veterinary Pathology, Free University Berlin, Robert von Ostertag Strasse 15, 14163 Berlin, Germany; ^4^ Mammal Research Institute, Department of Zoology and Entomology, University of Pretoria, Pretoria, Republic of South Africa

**Keywords:** hyperplasic spleen/splenomegaly, adaptive immune system, lymphopoiesis, extramedullary haematopoiesis, eusociality, naked mole-rat

## Abstract

Naked mole-rats (NM-R; *Heterocephalus glaber*) live in multi-generational colonies with a social hierarchy, and show low cancer incidence and long life-spans. Here we asked if an immune component might underlie such extreme physiology. The largest lymphoid organ is the spleen, which plays an essential role in responding to immunological insults and may participate in combating cancer and slowing ageing. We investigated the anatomy, molecular composition and function of the NM-R spleen using RNA-sequencing and histological analysis in healthy NM-Rs. Spleen size in healthy NM-Rs showed considerable inter-individual variability, with some animals displaying enlarged spleens. In all healthy NM-Rs, the spleen is a major site of adult haematopoiesis under normal physiological conditions. However, myeloid-to-lymphoid cell ratio is increased and splenic marginal zone showed markedly altered morphology when compared to other rodents. Healthy NM-Rs with enlarged spleens showed potentially better anti-microbial profiles and were much more likely to have a high rank within the colony. We propose that the anatomical plasticity of the spleen might be regulated by social interaction and gives immunological advantage to increase the lifespan of higher-ranked animals.

## Graphical abstract

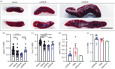

## 1. Introduction

Disease susceptibility is regulated by multiple factors including environmental stress and genetic factors. The immune system plays a critical role in protecting animals from infections and cancer. Optimal immune function is associated with healthy ageing [1,2]. In cases of pathogenic insult, the immune system protects the organism by engaging both innate and adaptive immune responses via either myeloid cells (granulocytes, macrophages and monocytes) and natural killer (NK) cells or lymphocytes and dendritic cells, respectively. Deregulation of the immune system is a critical factor in cancer and ageing as immune function declines with age [2].

Naked mole-rats (NM-Rs; *Heterocephalus glaber*) show an extraordinarily long lifespan for their small size (greater than 30 years) [3,4] and display a low cancer incidence [5–7]. Many features of NM-R physiology and habitat might contribute to the low cancer incidence, such as unique metabolic adaptations and hypoxia tolerance [8–10]. Recently, it has been shown that transformed NM-R cells can form tumours in mice [11], suggesting that non-cell autonomous mechanisms might eliminate tumorigenic cells before their spread in NM-Rs. Thus, the NM-R shows promise as an animal model to study the role of the immune system in cancer and ageing. NM-Rs are eusocial mammals that live in large colonies (on average 40–70 individuals), dominated by the queen, who is normally the only breeding female [12–14]. In our laboratory, we have kept NM-R breeding colonies for more than 10 years. Over the last 4 years we have been monitoring the health status and mortality of our NM-Rs, which rarely die in captivity. Indeed, we observed only one major cause of death, which was following fights with rivals during attempts to replace the breeding queen. Often, injured animals have unhealed infected wounds and have to be euthanized. *In vivo* experiments have shown that NM-Rs did not survive viral infections due to coronavirus or herpes simplex virus [15,16]. Single-cell-RNA sequencing analysis of the spleen and the peripheral blood of young adults showed that NM-Rs have a high myeloid to lymphoid cell ratio, but appear to lack classic NK cells [17]. These observations suggest that the NM-R immune system may differ significantly from that of conventional laboratory rodents.

In adulthood, secondary lymphoid organs like the spleen and lymph nodes participate in immune homeostasis. In humans and rodents, extramedullary haematopoiesis takes place in the spleen to support adult bone marrow haematopoiesis under stress conditions [18,19]. In addition, the spleen can also supply cells that stimulate cancer progression in mouse tumour models [20,21]. Hence, depending on the context, the spleen may support haematopoiesis, prevent the growth of cancer cells or facilitate the development of tumours in mice. In NM-R little is known about the structure and function of the spleen in normal physiological and pathological conditions. Here we investigated the role of the spleen in healthy NM-Rs using molecular profiling and anatomical analysis.

We show that the size of the spleen varies markedly between healthy NM-Rs, with higher-ranked animals displaying a larger spleen with pro-inflammatory features. NM-Rs with enlarged spleens did not show immature myeloid cells in the peripheral blood as observed in injured NM-Rs with wounds. In all healthy NM-Rs splenic and peripheral blood cell frequency showed an increased myeloid to lymphoid ratio, low bone marrow cellularity and extramedullary haematopoiesis taking place in the spleen with increased erythropoiesis, megakaryopoiesis and myelopoiesis, but reduced B lymphoid lineage compared to mice. B and T lymphocytes were found in secondary sites such as the lymph nodes, gut lymphoid sites and in the thymus, but the latter showed an unexpectedly reduced size in young adults. Our data suggest that, unlike other rodent species, the NM-R spleen is a major site of adult haematopoiesis under normal physiological conditions. However, the reduction in B lymphoid lineage suggests that NM-R immune system relies mainly on innate immune response with a more restricted adaptive immune response.

## 2. Results

### 2.1. Variable spleen size in NM-Rs

**Figure 1.**
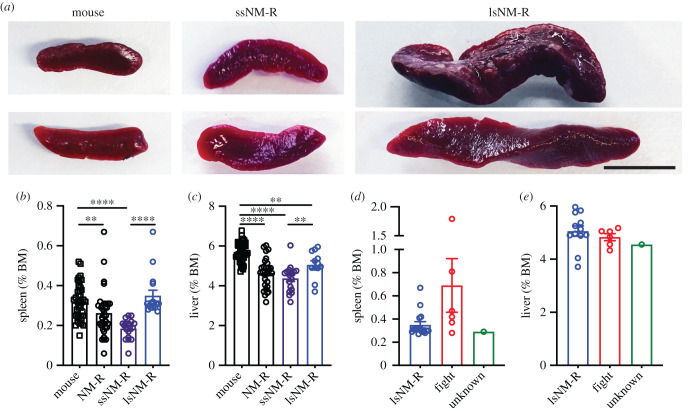
Variable spleen size in NM-Rs. (*a*) Representative images of NM-R small (ssNM-R) and large (lsNM-R) spleens compared to mouse spleens. Scale bar = 1 cm. (*b*) Spleen weight expressed as % of body mass (% BM) for mice (*n* = 40) and NM-Rs (*n* = 34 combined ssNM-R and lsNM-R, *n* = 18 ssNM-R, *n* = 16 lsNM-R). (*c*) Liver weight expressed as % BM for mice (*n* = 40) and NM-Rs (*n* = 30 combined ssNM-R and lsNM-R, *n* = 18 ssNM-R, *n* = 12 lsNM-R). (*d,e*) Comparison between spleen weight (*d*) and liver weight (*e*) of lsNM-Rs (same data as in (*b*,*c*)) and injured NM-Rs plotted per type of sickness (fighters *n* = 6 and unknown cause of sickness *n* = 1). Percent of BM (% BM) for each tissue type was calculated with BM and tissue weight in g. Graphs represent mean ± s.e.m. Unpaired *t* test: *p* value * < 0.05, ** < 0.01 and **** < 0.0001.

In order to study the structure and function of the NM-R spleen, we collected data from NM-R spleens over the last 4 years from a group of randomly sampled healthy animals (*n* = 34) aged between 1.3 and 5 years old, excluding breeding males and queens. Surprisingly, we observed that spleen mass and length varied considerably across healthy NM-Rs (figure 1*a*). Spleen size expressed as percentage of body mass (%BM) in C57BL/6N mice (*n* = 40, aged between 1 and 5 months) was on average 0.32% versus 0.26% in NM-Rs (*n* = 34). However, spleen size was much more variable in NM-Rs with healthy animals displaying very large or very small spleens (figure 1*a*). We divided the NM-Rs into two groups based on spleen size frequency distribution that showed a dip at around 0.25% of BM (electronic supplementary material, figure S1A). We classified NM-Rs according to spleen size, in the categories of small spleens (ssNM-R: %BM ≤ 0.26%) and large spleens (lsNM-R: %BM > 0.26%) (figure 1*b*). The mean spleen mass was 0.18% for ssNM-Rs and 0.35% for lsNM-Rs and the latter showed spleen masses similar to those of mice (figure 1*b*). Since the liver and the spleen can both be sites of extramedullary haematopoiesis and could become enlarged during infection or inflammation in rodents [18,19,22], we also measured liver mass (expressed as %BM) in the same NM-R cohort. Mean liver mass was slightly smaller in ssNM-Rs compared to NM-Rs with large spleens (figure 1*c*). NM-R livers (combined ssNM-R and lsNM-R) were significantly smaller compared to mice (figure 1*c*), but the liver size-frequency distribution showed a normal distribution in contrast to the spleen size-frequency distribution (electronic supplementary material, figure S1A, S1B). In NM-Rs the spleen size increased with age, while the liver mass was not affected by age (electronic supplementary material, figure S1C, S1E). In mice both organs decreased in size with age (electronic supplementary material, figure S1D, S1F). The mean age of ssNM-Rs was 29.7 ± 2.3 months versus 36.3 ± 2.9 months for lsNM-Rs which was not significantly different (unpaired *t*-test: *p* = 0.084). Spleen size was independent of sex in NM-Rs whereas female mice showed larger spleens compared to males (electronic supplementary material, figure S1G). Thus, the dynamics of spleen growth in young adult NM-Rs differed considerably from that of age-matched mice. Among the 34 NM-Rs, 29 were taken from three distinct colonies (named A, B and C), mean spleen mass was not different between colonies (electronic supplementary material, figure S1H).

### 2.2. Splenomegaly in lsNM-Rs is not associated with signs of infection

We next asked whether NM-Rs with enlarged spleens showed signs of ongoing illness or infection, as is the case for other rodents. Enlarged spleen (splenomegaly) may result from extramedullary haematopoiesis in the spleen and liver of individuals suffering from anaemia, neoplasia or myeloid hyperplasia in response to an infection or inflammation. Over a four-year period we collected the spleen and liver from seven sick NM-Rs. Among them, six animals were wounded from fighting and three of these animals had macroscopically infected wounds. These six animals served as positive controls for infection-associated splenomegaly (cohort named fight or NM-R fighters). One remaining animal showed signs of sickness, but of unknown cause. The injured NM-Rs that had engaged in fights showed the largest spleens (mean = 0.69% of BM) (figure 1*d*). The spleen size of NM-R fighters was twice the size of healthy NM-Rs (combined, mean = 0.26% of BM) or of the lsNM-R (mean = 0.35%) (figure 1*b,d*), indicating that splenomegaly does occur in NM-Rs following infection. There was no indication of enlarged livers in sick NM-Rs regardless of illness type (figure 1*e*).

**Figure 2.**
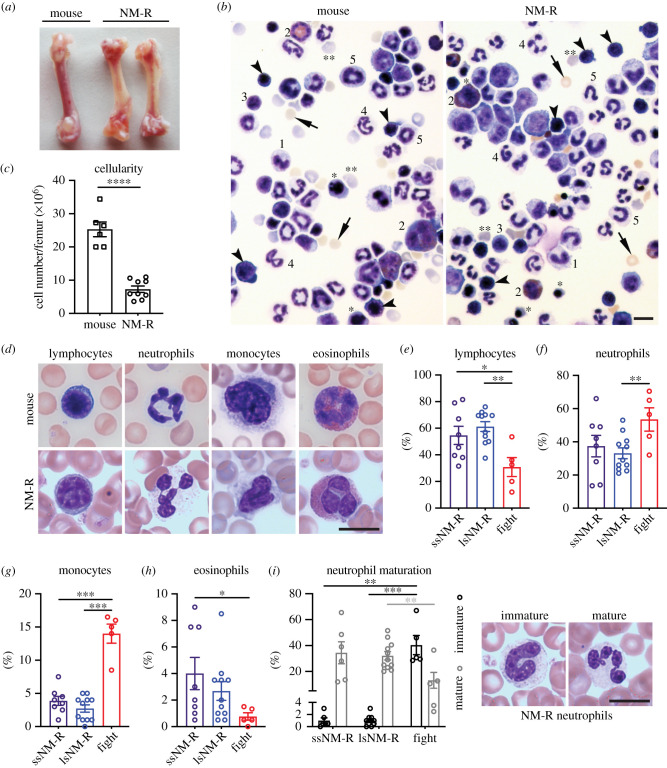
Wounded NM-Rs show high immature neutrophil count in peripheral blood unlike lsNM-Rs. (*a*) Representative images of femur from mouse and NM-R, (*b*) May–Grünwald staining of bone marrow cell cytospin without red blood cell lysis. Arrowhead: erythroblast; *orthochromatic erythroblast; **reticulocytes; arrow: mature erythrocytes; 1: monocytes; 2: eosinophils; 3: lymphocytes; 4: neutrophils; 5: band neutrophils. (*c*) Number of blood cells per femur, *n* = 6 mice, n = 9 NM-Rs. (*d*) Representative images of May–Grünwald staining of white blood cells in peripheral blood smears of NM-R and mice: lymphocytes, neutrophils, monocytes and eosinophils. (*e–h*) Percentage of lymphocytes (*e*), neutrophils (*f*), monocytes (*g*) and eosinophils (*h*) in the peripheral blood of NM-Rs using May–Grünwald staining of blood smears: *n* = 8 ssNM-R, *n* = 11 lsNM-R and *n* = 5 NM-R fighters. (*i*) Left panel: percentage of immature and mature neutrophils counted in blood smear of *n* = 8 ssNM-R, *n* = 11 lsNM-R and *n* = 5 NM-R fighters. Right panel: representative images of May–Grünwald staining of NM-R immature and mature neutrophils. All scale bars = 10 µm. Graphs represent mean ± s.e.m. Unpaired *t* test: *p* value * < 0.05, ** < 0.01, *** < 0.001 and **** < 0.0001.

In rodents and humans, increased numbers of immature myeloid progenitors and monocytes in peripheral blood are indicators of infection [23,24]. Since little is known about the blood cells of NM-R, we first examined bone marrow cells from healthy NM-Rs, the primary site of haematopoiesis in which haematopoietic stem cells generate all immune cells including erythroid, myeloid and lymphoid lineages. NM-R femurs were paler in colour than mouse femurs, suggesting lower haemoglobin and erythrocyte numbers (figure 2*a*). Cytospins of bone marrow cells that were not subjected to erythrocyte lysis indicated that all cell types of the erythroid lineage including mature erythrocytes, reticulocytes, orthochromatic erythrocytes and erythroblasts are present in the bone marrow of the NM-R (figure 2*b*). In addition, all known haematopoietic cell types found in mouse bone marrow were also present in the NM-R including myeloid and lymphoid lineages (figure 2*b*). Surprisingly, in NM-Rs the immature neutrophils (also called band neutrophils) are stab-cell-shaped, similar to those of humans [24], while characteristic ring-shaped neutrophils of the mouse and rat were not found (figure 2*b*). Furthermore, the cell number was 3 times lower in NM-R femur compared to mouse femur (7.3 × 10^6^ in NM-R versus 25 × 10^6^ in mouse, figure 2*c*). Bone marrow haematopoietic cells differentiate and are found in the peripheral blood from where they can be further characterized. We, therefore, analysed 23 NM-R blood samples from healthy animals (*n* = 11 from ssNM-Rs and *n* = 12 from lsNM-Rs) using an automated blood counter and mouse blood as reference (electronic supplementary material, figure S2A–S2E). We found similar total white blood cell counts in both species (electronic supplementary material, figure S2A), but the frequency of the various cell populations was altered with an increased neutrophil/lymphoid ratio and a higher number of monocytes in the peripheral blood of NM-Rs (electronic supplementary material, figure S2B–S2D). The eosinophil count was only modestly increased (electronic supplementary material, figure S2E). These blood counts were similar in NM-Rs with small and large spleens, with ssNM-Rs showing slightly lower white blood cell counts (electronic supplementary material, figure S2A). Thus, our data indicate that the bone marrow of NM-Rs can give rise to all cell types described in mice and humans, but the distribution of the haematopoietic cells in peripheral blood was more similar to that of humans with increased circulating myeloid cells at the expense of circulating lymphocytes.

These data suggested that lsNM-Rs with enlarged spleens are healthy. To verify this, we examined in detail monocytes and immature neutrophil populations in the peripheral blood of animals with infection or inflammation (NM-R fighters) and compared them to those from lsNM-Rs. Analysis of May–Grünwald stained blood smears from 19 NM-R healthy animals (*n* = 8 ssNM-Rs and *n* = 11 lsNM-Rs) confirmed blood counter data (figure 2*d–h*; electronic supplementary material, figure S2B–S2E). Interestingly, blood smears from NM-R fighters (*n* = 5) showed dramatic increases in the monocyte population and a reduction in lymphocytes compared to all NM-R cohorts (figure 2*e,g*). In addition, 53% of the white blood cells were immature neutrophils (band neutrophils) and 13% were segmented neutrophils (mature stage) in the peripheral blood of the NM-R fighters, indicating an active immune response against infection or inflammation (figure 2*i*). By contrast, almost exclusively mature neutrophils (segmented neutrophils: 33 to 41% of white blood cells versus ≤ 1% of immature neutrophils) were found in healthy NM-Rs (ssNM-R and lsNM-R) (figure 2*i*). Our results clearly demonstrate that NM-Rs with apparent splenomegaly do not show myeloid hyperplasia like injured NM-Rs.

### 2.3. Increased myeloid and reduced lymphoid lineages in NM-R spleen

**Figure 3.**
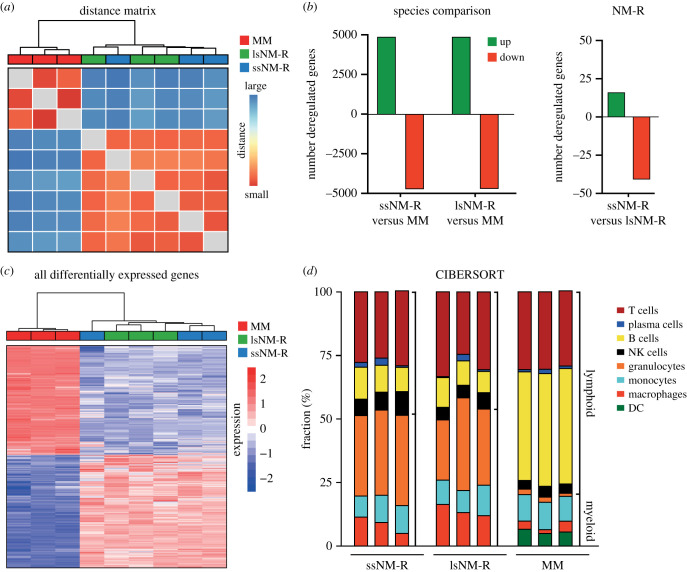
Transcriptomic analysis of NM-R and mouse spleen. (*a*) Heatmap visualization of the Euclidean distance between spleen samples based on global transcriptomic data (RNAseq). (*b*) Number of differentially regulated genes (upregulated in green, downregulated in red) in the spleens of ssNM-R, lsNM-R compared to mouse (MM, left panel) or compared to each other (right panel). (*c*) Heatmap of all differentially expressed genes in the spleen of ssNM-Rs and lsNM-Rs in comparison to mouse. Samples are hierarchically clustered based on Pearson correlation. (*d*) Fractions of major splenic cell types based on *in silico* cytometric analysis of transcriptomic data (CIBERSORT). DC: dendritic cells, NK: natural killer cells, MM: mouse (*Mus musculus*); *n* = 3 per group.

The differences in spleen size found in healthy NM-R cohorts might reflect specific cell type hyperplasia between both cohorts. To address this, we applied global gene expression profiling to investigate molecular differences between small and large NM-R spleens compared to the mouse. We also compared RNAseq data from mouse spleens with NM-R in order to reveal whether NM-R and mouse spleen share molecular signatures. Global comparison of transcriptomes (including transcripts from 12 946 genes) indicated major differences between the two species and high similarity between ssNM-R and lsNM-R (figure 3*a*). Principal component analysis and Venn diagram analysis also showed clear species differences and only loose clustering of large and small NM-R spleens (electronic supplementary material, figure S3A,B). Differential gene expression analyses of the three groups showed 4869 and 4873 upregulated genes and 4737 and 4713 downregulated genes in ssNM-R and lsNM-R, respectively, compared to mice (figure 3*b,c*). In ssNM-R spleens just 16 genes were differentially upregulated and 41 were downregulated compared to lsNM-R spleens (figure 3*b*). Comparing NM-R with mouse spleen the RNAseq data revealed a dramatic reduction in the expression of B cell markers (such as *Cd19*, *Cd79a* and *Cd79b*), and of dendritic cell markers such as *Itgax* (coding for CD11c), but there was an increase in the expression of myeloid markers such as *Itgam* (coding for CD11b) and a modest increase in monocytic/macrophage gene expression (*Cd14*) (electronic supplementary material, figures S3C and S3D, and table S1). To infer immune cell type abundance in the spleen from the bulk RNAseq data digital cytometry using CIBERSORT analysis was performed [25]. Since no pre-defined signatures exist for immune cell subsets of NM-Rs, mouse gene expression signatures for the cell subsets were used [25,26]. The analysis predicted that 53 ± 3% of myeloid cells (31.8 ± 4.6% granulocytes, 10 ± 1% monocytes and 11 ± 4% macrophages), 48 ± 3% of lymphoid cells (10 ± 4% B cells, 2 ± 1% plasma cells, 7 ± 2% activated NK cells and 29 ± 3% T cells) are present in NM-R spleens regardless of size (figure 3*d*; electronic supplementary material, figure S3E). This shift toward myeloid cells was highly consistent with recently published single-cell RNAseq profiling data from the NM-R [17]. In addition, our functional analysis using gene set enrichment analysis (GSEA) highlighted a significant underrepresentation of gene sets implicated in B cells homeostasis, regulation and proliferation (electronic supplementary material, tables S2 and S3). Of note, the percentage of total T-cells was similar in both species, but in NM-Rs the T-cell subset distribution differed from that of mice, including the presence of gamma-delta T-cells and Th1 cells (electronic supplementary material, figure S3E, left panel). A significant change in the expression of T cell-associated gene sets by GSEA was found (electronic supplementary material, tables S2–S4). Thus, the data suggested that lymphopoiesis and myelopoiesis are differently regulated in adult NM-Rs compared to mice, predicting a special role for the spleen in this species.

### 2.4. The NM-R spleen has unique structural features

**Figure 4.**
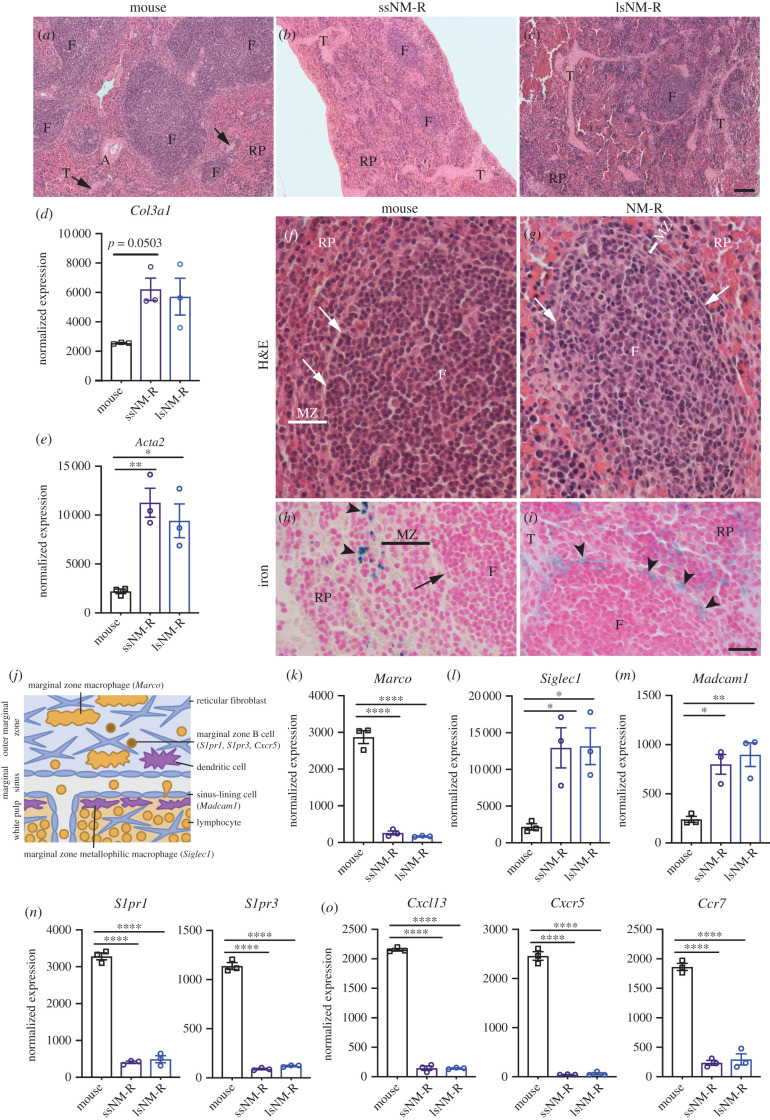
NM-R spleen shows a reduced white pulp compartment and a thin marginal zone. (*a–c*) H&E staining of the spleen of ssNM-R (*b*) and lsNM-R (*c*) in comparison to mouse (*a*). Note the increase in red pulp/white pulp ratio with reduced number and size of follicles (F) and increased number of trabeculae (T, arrow) in NM-R spleens. (*d–e*) Increase in normalized RNA expression levels of *Col3a1* (*d*) and of *Acta2* (*e*) in NM-R spleens compared to mouse. (*f–g*) H&E staining of mouse (*f*) and NM-R (*g*) spleen showing the presence of follicles (F) with a thinner marginal zone (MZ) in NM-R compared to mouse. (*h–i*) Iron staining of mouse (*h*) and NM-R (*i*) spleen showing stained macrophages (blue iron staining, arrowhead) close to the follicle in NM-R but not in mouse marginal zone. MZ: marginal zone (black or white line), RP: red pulp, marginal sinus (arrow in *f–h*). (*j*) Schematic representation of the mouse marginal zone and its cell types with their expression markers. (*k–o*) Normalized RNA expression levels of *Marco* (MZ macrophage marker) (*k*), of *Siglec1* (MZ metallophilic macrophage marker) (*l*), of *Madcam1* (sinus lining cells marker) (*m*), of markers of MZ B cells (*S1pr1, S1pr3*) (*n*) and of chemokine-chemokine receptors involved in the lymphocytes migration into the white pulp (*Cxcl13, Cxcr5 and Ccr7*) (*o*). One-way ANOVA with Tukey's *post hoc* test for multiple comparisons: *p* value *<0.05, **<0.01 and ****<0.0001. Data is based on RNAseq and bars represent mean ± s.e.m. Scale bar = 100 µm (*a–c*) and 20 µm (*f–i*).

The spleen is composed of two functionally and morphologically distinct compartments, the white pulp and the red pulp. The white pulp contains most of the lymphocytes and initiates the immune responses to blood-born antigens while the red pulp is a blood filter that removes foreign material and damaged or senescent erythrocytes, and is a storage site for iron, erythrocytes and platelets [27]. We predicted that the loss of 30–40% of splenic lymphocytes and the 50% increase in myeloid cells would impact the structure and function of the spleen. Indeed, histological analysis of NM-R spleens showed a strongly reduced white pulp volume and an increase in trabeculae abundance that was independent of spleen size (figure 4*a–c*). We wondered whether these structural peculiarities were observed in other African mole-rat species. We had access to spleens from two other Bathyergidae species the Natal and Highveld mole-rats (*Cryptomys hottentotus natalensis* and *Cryptomys hottentotus pretoriae*) [28,29] that are related to NM-Rs but are not eusocial mammals (electronic supplementary material, figure S4A). The structure of the NM-R spleen did not resemble that of spleens from Natal and Highveld mole-rats, which were both very similar to the mouse and rat (figure 4*a*; electronic supplementary material, figure S4B–D). The increased trabeculae density in NM-R spleens was accompanied by a small increase in the expression of the *Col3a1* gene (coding for Type III collagen) a reticulin fibrin component and by a 5-fold increase in the RNA level of α-SMA (encoded by *Acta2* gene) (figure 4*d,e*). These two genes may be associated with the fibrous trabeculae that act as a pump to filter blood.

The white pulp consists of three sub-compartments: the periarteriolar lymphoid sheath (the T cell zone), the follicles and the marginal zone [27]. In NM-R spleens the follicles were very small and reduced in number compared to mouse spleen (figure 4*a–c*) and were surrounded by a very thin marginal zone (figure 4*f,g*). The marginal zone is where the blood is filtered from pathogens and is organized in layers with the marginal zone macrophages, the reticular fibroblasts and marginal zone B cells all facing the red pulp. The marginal sinus with its sinus lining endothelial cells and an inner ring of marginal zone metallophilic macrophages separate the marginal zone from the periarteriolar lymphoid sheath and follicles (figure 4*j*) [30]. Iron staining labelled red pulp macrophages in mice that are localized to the red pulp (figure 4*h*). In NM-Rs iron-stained macrophages were found not only in the red pulp but also close to the follicles (figure 4*i*), suggesting a microarchitectural change of the marginal zone. These anatomical changes were reflected in our RNAseq data that showed decreased *Marco* expression (10-fold compared to mouse), a marker of marginal zone macrophages (figure 4*k*), suggesting reduced abundance or loss of marginal zone macrophages. By contrast, there was a 3-fold increase in the expression of marginal zone metallophilic macrophage and sinus lining markers (*Siglec1* and *Madcam1*, respectively) compared to mice (figure 4*l* and *m*). The expression of marginal zone B cell receptors (*S1pr1, S1pr3, Cxcr5*) involved in marginal zone B cell migration to the follicles were also decreased probably due to the reduced abundance of marginal zone B-cells (figure 4*n* and *o*). Taken together, the low number of marginal zone B cells and marginal zone macrophages could explain the altered morphology of the NM-R marginal zone. This microarchitectural change of the marginal zone might contribute to impaired adaptive immunity, in particular the proper binding and clearance of blood-borne pathogens. In addition, we found that enlarged spleens of lsNM-Rs did not show signs of pathology associated splenomegaly (figure 4*c*).

### 2.5. Increased splenic granulocytes at the expense of the lymphoid compartment

**Figure 5.**
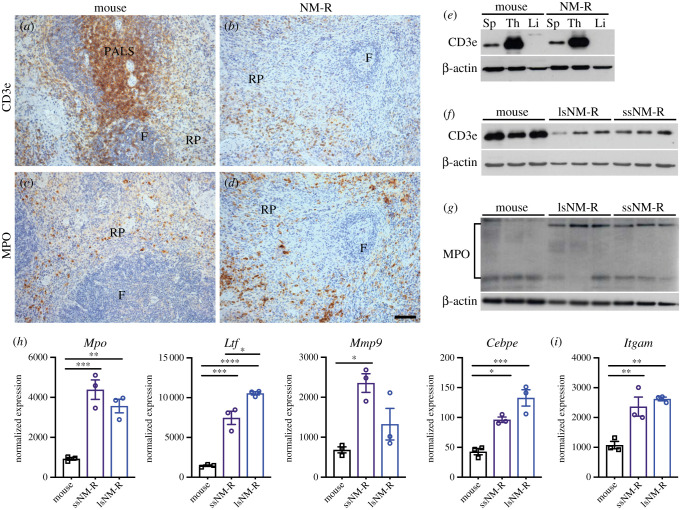
The granulocyte population is increased in NM-R spleen. (*a,b*) Immunostaining of the splenic T-cells with CD3e antibody and of the splenic granulocytes with myeloperoxidase (MPO) antibody (*c,d*) in NM-R and mouse. (*e*) Protein expression of CD3e in various tissues of NM-R and mouse: Sp: spleen, Th: thymus, Li: liver. (*f*) CD3e (T-cells marker) protein expression in three spleens of mice, lsNM-Rs and ssNM-Rs. (*g*) MPO (granulocyte marker) protein expression in three spleens of mice, lsNM-Rs and ssNM-Rs. (*h*) Increase in normalized RNA expression levels of granulocyte markers (*Mpo, Ltf, Mmp9* and *Cebpe*) and (*i*) myeloid marker (*Itgam*) in NM-R spleens in comparison to mouse spleens. β-actin expression was used as loading control in (*e*,*f*,*g)*. F: follicles, PALS: periarteriolar lymphoid sheath, RP: red pulp. Data in (*h*) and (*i*) are based on RNAseq and bars represent mean ± s.e.m. One-way ANOVA with Tukey's *post hoc* test for multiple comparisons: *p* value * < 0.05, ** < 0.01, *** < 0.001 and **** < 0.0001. Scale bars = 50 µm.

The formation and maintenance of follicles in lymphoid tissues such as the spleen are regulated by chemokines and their cognate receptors expressed by stromal cells to generate a microenvironment necessary for B and T cell homing to the follicles [31]. The expression levels of chemokine genes (*Cxcl13* and *Ccl19*) and their respective receptors (*Cxcr5 and Ccr7*) were decreased in NM-R spleen compared to mice (figure 4*o*, electronic supplementary material, table S1). Using the T cell marker CD3e we could show that in NM-Rs T-cells were mainly present in the red pulp, but in much smaller numbers than in mice (figure 5*a,b*). In both species, western blot analysis showed higher expression of CD3e in thymus (site of T lymphopoiesis) compared to spleen, while no expression was found in the liver, a non-immunological organ (figure 5*e* and electronic supplementary material, figure S10A). Furthermore, CD3e expression was lower in NM-R spleens regardless of their size compared to mouse (figure 5*f* and electronic supplementary material, figure S10B). Unfortunately, we could not confirm the decrease in B cells in NM-R spleens using immunostaining because of the lack of NM-R specific reagents, but H&E staining rarely showed the presence of follicles and germinal centres, both structures harbouring B cells. These data suggest that splenic adaptive immune responses may rely mainly on T cells in the NM-R.

Our transcriptomic analysis also predicted increased myeloid populations in particular of granulocytes in NM-R compared to mouse (31.8 ± 4.6% versus 1.8 ± 0.5% total granulocytes including 29.2 ± 4.6% versus 1.4 ± 0.4% neutrophils, and 2.3 ± 1.3% versus 0.4 ± 0.3% eosinophils in NM-R (*n* = 6) versus mouse (*n* = 3), respectively) (figure 3*d*). This was supported by immunohistochemistry and western blot analyses using an antibody directed against myeloperoxidase (MPO) a marker of pre-and mature granulocytes. We also found higher protein levels of MPO in the splenic red pulp of NM-Rs compared to mice (figure 5*c,d,g* and electronic supplementary material, figure S10C). The expression levels of *Mpo* and 3 other markers of granulocytes, *Ltf* (granules of neutrophil granulocytes), *Mmp9* and *Cebpe* (major transcription factor of neutrophil lineage) were increased in NM-R spleens, demonstrating that NM-Rs have more splenic granulocytes than mice independent of size (figure 5*h*). Of note, the RNA level of the common marker of myeloid cells, *Itgam* (coding for CD11b) was also increased (figure 5*i*). In summary, our data suggest that NM-Rs have enhanced antimicrobial innate immunity, but their adaptive immunity might be less efficient compared to mice.

### 2.6. Lymphocytes locate to peripheral blood and lymphoid tissues in NM-Rs

Gene expression profiling and histological analysis consistently showed that NM-Rs have almost 50% fewer splenic resident lymphocytes compared to other rodents. This unique immunological feature might have major consequences for adaptive immunity unless compensatory lymphopoiesis occurs in the bone marrow or other secondary lymphoid organs. Lymphocytes were found in bone marrow cytospins and in peripheral blood, but at much lower frequencies than in mice (figure 2). By contrast to other bone marrow haematopoietic progenitors that undergo several differentiation stages before egression and maturation, T-lymphocyte progenitors migrate to the thymus to differentiate into naive T-cells that can migrate to the blood and secondary lymphoid organs. Intriguingly, in young adult NM-Rs the thoracic thymus was often embedded in brown adipose tissue (electronic supplementary material, figure S5A and S5B bottom) and the thoracic thymus/body mass ratio was considerably lower in NM-Rs compared to young C57BL/6N mice that do not yet show thymus involution (electronic supplementary material, figure S5B top). Histology of the thoracic thymus revealed a clear cortex and medulla (electronic supplementary material, figure S5C) in which the naive T lymphocyte marker CD3e was highly expressed in both mice and NM-Rs (electronic supplementary material, figure S5D). The NM-R thoracic thymus contains CD3e+ T cells, but its small size prompted us to search for other sites of lymphopoiesis.

Lymphocytes (T and B cells) are also found in lymph nodes. We next analysed NM-R axillary lymph nodes that are 2–4 millimetres in length, similar to those of mice (electronic supplementary material, figure S6A top panel). Lymph nodes are structurally organized in B and T cell areas, a process regulated by cytokine signalling (electronic supplementary material, figure S6A, bottom panel). Histologically, B cell and T cell areas were easily identified in axillary and mesenteric lymph nodes of NM-Rs and B cell areas possessed germinal centres (electronic supplementary material, figure S6B). T cell areas showed high expression of CD3e (electronic supplementary material, figure S6C).

Since half of the lymphocytes are located in the mucosa-associated lymphoid tissue in mice, we next focused on lymphoid nodules of the small intestine including among others Peyer's patches [32]. Peyer's patches are visible to the naked eye in mouse small intestine but were rarely apparent in NM-R gut (electronic supplementary material, figure S6D,E). In addition, the small intestine of NM-Rs was only a third of the length of that in the mouse, however, the length of the colon was similar (electronic supplementary material, figure S6D,F–G). Histological analysis of the NM-R small intestine showed lymphoid nodules with a morphology atypical for Peyer's patches, but these follicles displayed B cell areas with germinal centres containing apoptotic cells and T cell areas expressing CD3e in both NM-Rs and mice (electronic supplementary material, figure S6H,I). In general, we found that NM-Rs and mice have similar lymph nodes with well-structured B and T cell areas, but unlike in mice, the NM-R small intestine did not appear to have typical Peyer's patches, even in injured animals (electronic supplementary material, figure S6E).

### 2.7. Increased extramedullary erythropoiesis is likely not a cause of splenomegaly

**Figure 6.**
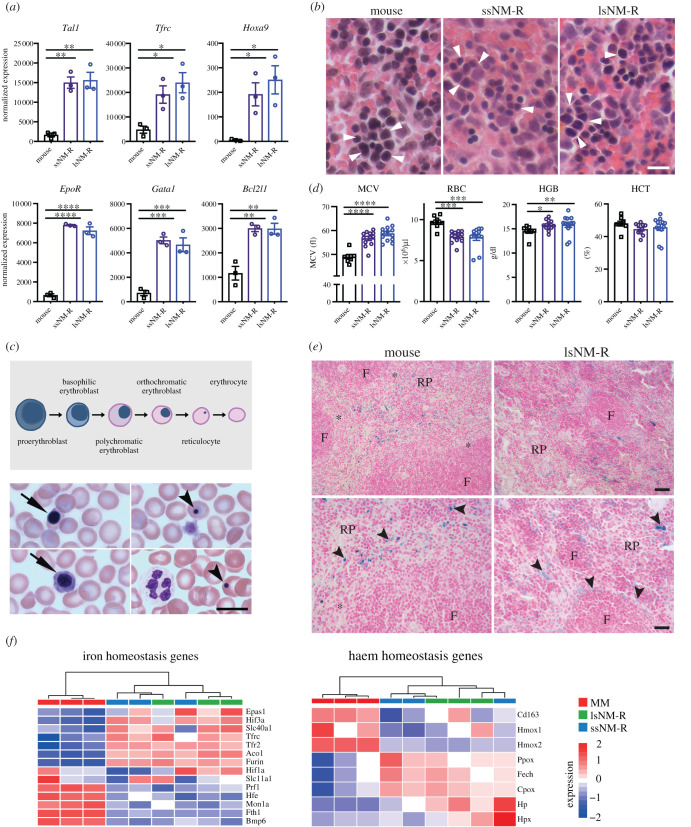
Extramedullary erythropoiesis and iron homeostasis do not explain the hyperplasic spleen of lsNM-Rs. (*a*) Normalized RNA expression levels of early erythroid progenitors (*Tal1*, *Tfrc*, *Hoxa9*) and of erythroid precursor proliferation and survival (*EpoR*, *Gata1*, *Bcl2l1*) markers in mouse and NM-R spleens. (*b*) Representative micrographs of H&E staining of erythroid cells (arrowheads) in the spleen of mouse, ssNM-R and lsNM-R. (*c*) Schematic representation of late erythroid lineage (top panel). Representative May–Grünwald staining of orthochromatic erythroblast (arrow) in peripheral blood of NM-R, Howell–Jolly bodies in reticulocytes (arrowhead) in bottom panel. (*d*) Peripheral blood erythroid parameters in mice (*n* = 8), ssNM-R (*n* = 13) and lsNM-R (*n* = 13). MCV: mean corpuscular volume, RBC: red blood cells, HGB: haemoglobin, HCT: haematocrit. (*e*) Perl's Prussian blue staining of ferric iron (arrowhead) in the spleen of NM-Rs and mice. Macrophages close to follicles (F) and in red pulp (RP) contain ferric ion in NM-Rs while in mice only RPM contained ferric ion. Marginal zone: asterisk. (*f*) Heatmap of genes associated with iron and haem homeostasis in NM-R and mouse spleen. Samples are hierarchically clustered based on Pearson correlation. One-way ANOVA with Tukey's *post hoc* test for multiple comparisons (*a*) and unpaired *t* test (*d*): *p* value * < 0.05, ** < 0.01, *** < 0.001 and **** < 0.0001. Transcriptomic data is based on RNAseq, MM: mouse (*Mus musculus*). Data represent mean ± s.e.m, scale bar = 10 µm (*b,c*), 20 µm (*e* bottom panels) and 40 µm (*e* top panels).

The unique structural features of the NM-R spleen could not account for the hyperplasic phenotype of the spleen found in the lsNM-R cohort. In mice, splenomegaly can be observed under erythropoietic stress such as hypoxia [33,34]. Since hypoxia is a normal environmental condition for NM-Rs we hypothesized that extramedullary erythropoiesis might occur naturally in NM-R spleen, but more actively in lsNM-R spleens. RNAseq analysis and GSEA highlighted a significant enrichment in erythroid gene subsets (electronic supplementary material, figure S7A, S7B), including markers of early erythroid progenitors such as *Tal1* (erythroid differentiation factor), *Tfrc* (coding for CD71), *Hoxa9* (a marker of pro-erythroblast and basophilic erythroblast) and markers of erythroid precursor proliferation or survival such as *EpoR* (the Epo receptor), *Gata1* and *Bcl2l1* (coding for Bcl-XL) [35–38] (figure 6*a*). Thus, early erythroid progenitors appeared more abundant in NM-R spleen compared to mice, regardless of spleen size. Histological analysis revealed erythroid cells in mouse and NM-R spleens often organized in erythroid blood islands (figure 6*b*). Immature erythrocytes (enucleated red blood cells also called orthochromatic erythroblasts, see the schematic representation of the erythroid lineage in figure 6*c*, top panel) were present in the peripheral blood of NM-Rs (figure 6*c*, bottom panel), but not in healthy mice. In addition, mature erythrocytes (RBC) were larger in NM-Rs as indicated by a higher mean corpuscular volume (MCV), but contained less haemoglobin (HGB) and were less abundant in peripheral blood compared to mice (figure 6*d*). However, the haematocrit (HCT) was not different between mouse and NM-R (figure 6*d*). Altogether our results showed that extramedullary erythropoiesis occurs in the spleen of both ssNM-Rs and lsNM-Rs, but cannot account for splenomegaly in lsNM-Rs.

### 2.8. Iron homeostasis is not a cause of splenomegaly in lsNM-R

Thus, NM-Rs probably adapted erythropoiesis to their unusual environmental conditions. The expression levels of several known hypoxia-induced genes (*EpoR, Tfrc, Tfr2, Furin*) were increased compared to the mouse (figure 6*a*; electronic supplementary material, figure S7A and table S1), but GSEA analysis did not highlight significant changes in hypoxia-regulated gene subsets (electronic supplementary material, tables S2 and S3). In humans and mice erythropoiesis is not only regulated by oxygen availability, but also depends on intracellular iron levels. Indeed, genetic defects in iron or haemoglobin metabolism can lead to splenomegaly in mice [39,40]. Iron staining was found as expected in red pulp macrophages, cells responsible for efficient phagocytosis of red blood cells and storage of iron [41]. The overall iron accumulation was similar in NM-R spleens independent of their size compared to mouse (figure 6*e*). Intriguingly, we found that the overall GSEA red pulp macrophage gene subset was significantly increased in NM-R compared to the mouse (electronic supplementary material, figure S7C, and table S4), but RNA levels of *SpiC, Adgre1* (coding for F4/80) and *Cd68*, classical phenotypic markers of mouse red pulp macrophages [42], were strongly decreased in both small and large NM-R spleens (electronic supplementary material, figure S7D). Lastly, our GSEA analyses showed no significant changes in gene subsets implicated in iron and haem homeostasis (figure 6*f*; electronic supplementary material, tables S1–S3). As a consequence, our data suggest that naturally occurring splenomegaly was not due to defective iron homeostasis.

### 2.9. Extramedullary megakaryopoiesis does not account for splenomegaly

The erythroid lineage shares a common progenitor with megakaryocytes, the so-called megakaryocyte-erythroid progenitors (MEP) which give rise to erythroid and megakaryocyte lineages in bone marrow (electronic supplementary material, figure S8A). We hypothesized that extramedullary megakaryopoiesis might also occur in the NM-R spleen. RNAseq analysis indicated that expression of many megakaryocyte and platelet genes are upregulated in NM-Rs compared to mice independent of spleen size (electronic supplementary material, figure S8B). Megakaryocyte differentiation occurs in several steps from MEP to activated megakaryocytes that release platelets into the peripheral blood [43] (electronic supplementary material, figure S8A). The RNA levels of marker genes of MEP and early megakaryocytes (*Cd34*, *Gfi1b*, *Fli1*, *Itga2b*), terminal differentiation genes (*Nfe2*, *Tubb1*) and platelets (*Gp1bb*, *Itgb3*, *Cd63*) were all elevated in NM-R spleens compared to that of mice (electronic supplementary material, figure S8B). Histological analysis also showed a significant increase in the number of megakaryocytes per unit area in lsNM-Rs compared to the rat spleen and a slight increase in megakaryocyte number in NM-R (combined) compared to those of the mouse and rat (electronic supplementary material, figure S8C,D). Surprisingly, blood counts indicated that platelets were less abundant in the peripheral blood of all NM-R cohorts compared to mice (electronic supplementary material, figure S8E), but NM-R platelet size was larger as shown by mean platelet volume (electronic supplementary material, figure S8F). Blood smear analysis showed the presence of immature and mature platelets in NM-R peripheral blood, whereas only mature platelets were observed in mice (electronic supplementary material, figure S8G). Intriguingly, the expression levels of regulatory genes of megakaryocyte terminal differentiation (*Ccl5*, *Il1a* and *Igf1*) were reduced compared to the mouse (electronic supplementary material, figure S8H). Taken together our data suggest that megakaryocytic differentiation occurs efficiently in NM-Rs, but the terminal differentiation step(s) might be regulated differently. The presence of immature platelets in peripheral blood of NM-Rs might reflect a thrombocytopenia-like phenotype as observed in thrombocytopenia or inherited diseases in humans [44].

### 2.10. Hyperplasic spleens are associated with higher rank

**Figure 7.**
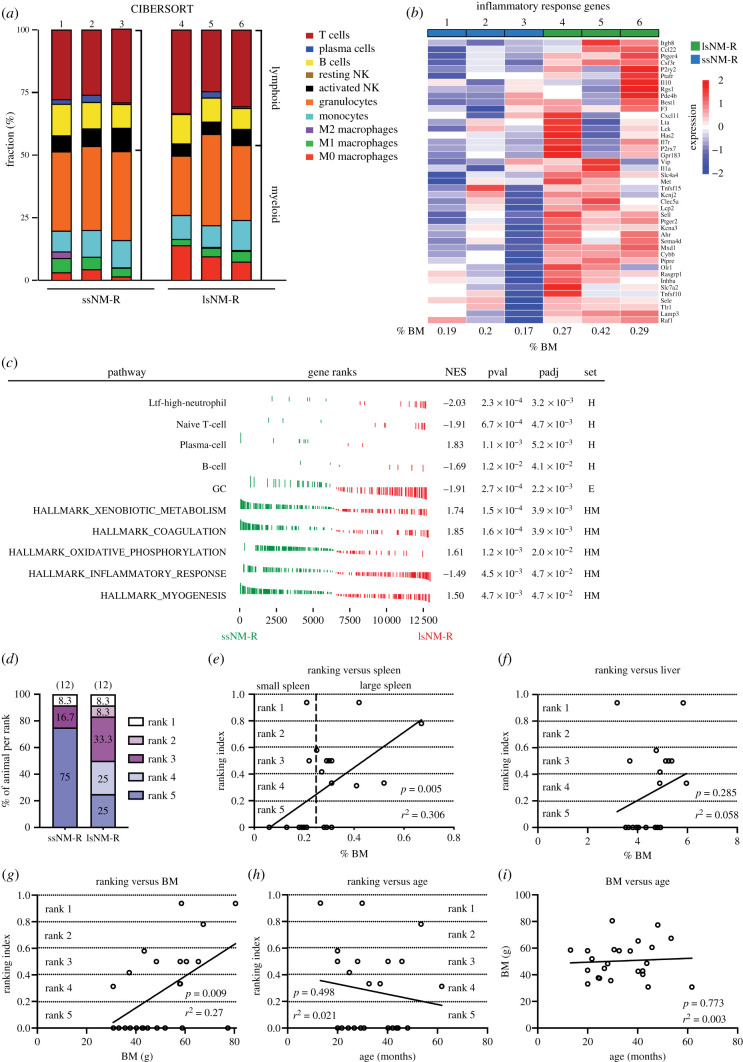
Hyperplasic spleens in lsNM-R are associated with higher rank. (*a*) Relative fraction of immune cells predicted by CIBERSORT in ssNM-R and lsNM-R spleens (*n* = 3 per cohort). NK: natural killer cell. (*b*) Heatmap representation of leading edge inflammatory response genes for ssNM-R and lsNM-R spleens identified by hallmark GSEA analysis. (*c*) GSEA of transcriptomic data from ssNM-R and lsNM-R spleens (*n* = 3 per cohort). NES, normalized enrichment score; pval, *p* value; *p*_adj_, adjusted *p* value. Significantly enriched pathways using NM-R cell-type signatures from Hilton and colleagues (H) [17], Emmrich and colleagues (E) [72] and established hallmark pathways (HM). GC: granulocytes. (*d*) Percentage of animal per rank and per cohort. Note that only 25% of the lsNM-R has the lowest ranking index (rank 5) compared to 75% in ssNM-R. (*e–h*) Correlation between rank of NM-R in their colony (ranking index) and spleen size (%BM) (*e*), liver size (%BM) (*f*), body mass (BM) (*g*) or age (*h*), showing that the ranking index of NM-Rs positively correlated with their spleen size and BM but not with their liver size and age. (*i*) Body mass and age of NM-Rs poorly correlated). Simple linear regression analysis was used to test goodness of fit: the calculated r square (*r*^2^) and *p*-value are given for each line. NM-Rs *n* = 24 including *n* = 12 ssNM-R, *n* = 12 lsNM-R.

We detected a small set of differentially expressed genes between NM-Rs with large and small spleens (41 upregulated genes and 16 downregulated) (figure 3*b*; electronic supplementary material, figure S9). Prediction of immune cell distribution using CIBERSORT analysis showed a specific difference in the apparent incidence of the M0 macrophage subtype (2.8 ± 1.5% in ssNM-R versus 10.4 ± 3.4% in lsNM-R) (figure 7*a*). Furthermore, GSEA highlighted significant increases in hallmark gene subsets such as inflammatory response, granulocytes, naive T- and B-cell pathways, and the Ltf-high-neutrophil subset in lsNM-R compared to ssNM-R (figure 7*b,c*). These findings suggested that NM-Rs with larger spleens are better equipped for defence against pathogens. Indeed, larger spleen size might confer a survival advantage for NM-Rs. NM-Rs are eusocial mammals with a structural hierarchy with the queen and her consorts occupying the highest rank [12]. We modified a ranking index of animals in a colony with the highest ranking set to 1 for the queen and the lowest to 0 for subordinate [13,45]. The ranking index of 24 healthy NM-Rs (12 ssNM-R, 12 lsNM-R) used in this study had been determined (figure 7*d*). Strikingly, most of the animals (75%) with a small spleen were found to have the lowest rank (figure 7*d*). By contrast, many more of the animals with large spleens belonged to the higher ranks (figure 7*d*). We found a significant positive correlation between ranking index and spleen mass (%BM) when combining all healthy cohorts (ssNM-R, lsNM-R) (figure 7*e*). By contrast, the liver mass (%BM) was poorly correlated with the ranking index in the combined healthy cohort (figure 7*f*). There was a significant correlation between rank and BM (figure 7*g*), but the age of the animals was a poor predictor of ranking or BM (figure 7*h,i*). Our data identify rank as being predictive of spleen size in healthy animals with large spleens probably conferring an immunological advantage over lower-ranked animals.

## 3. Discussion

An enlarged spleen may indicate that the immune system is reacting to infection or inflammation in rodents [18,19]. In our survey of the immune system of NM-Rs we found an unusual variation in spleen size in apparently healthy individuals. Healthy animals with enlarged spleens showed similar white blood cell composition and splenic structural features to animals with small spleens. By contrast, NM-Rs suffering from wound infection displayed enlarged spleens with accompanying signs of immune activation like increased blood monocytes and increased numbers of immature neutrophils (figure 2*g,i*). Interestingly, the spleens of injured animals were on average larger than those of lsNM-Rs, but this was not statistically different (figure 1*d*). We could show that in healthy animals both small and enlarged spleens were associated with enhanced erythropoiesis, megakaryopoiesis and myeloid hyperplasia when compared with mice (figures 5 and 6; electronic supplementary material, figure S8). However, we detected significant molecular differences between small and enlarged spleens of healthy NM-Rs, with large spleens harbouring larger numbers of an LPS-responsive granulocyte population (also called Ltf-high-neutrophil), recently described in NM-Rs [17]. Intriguingly, the molecular profile of larger spleens suggested a pre-activated state that might prepare the animal to better fight infection. One unique feature of NM-R colonies is that they display a hierarchical structure, with the highest-ranked members most likely to be or become breeders [12]. Indeed, it has also been shown that higher-ranked NM-Rs such as breeders show longer lifespans compared to lower-ranked individuals [4] as well as better survival rates following viral infection compared to non-breeders [15]. Furthermore, higher-ranked individuals are often tasked with colony defence, hence these individuals have a higher risk of coming into contact with intruders which may carry pathogens [12]. Body mass is positively correlated with rank in NM-Rs [13,45], and here we extend this finding by showing that spleen size also positively correlates with the rank of animals. Interestingly, the size of other organs like the liver showed no correlation with the animal's rank. We propose that NM-Rs with enlarged spleens may have a survival advantage over lower-ranked animals. The immunological repertoire of animals with large spleens may help them to better fight infection, or could even confer cancer resistance. Thus, we have shown a remarkable plasticity in the immune system of NM-Rs that may be regulated through social interaction. When members of the colony get sick, social distancing, as practised by some species [46], may not be feasible. Thus, tuning of immune competence in higher-ranked NM-Rs may be a novel strategy in the animal kingdom to deal with the challenge of infection in a tightly knit colony. The factors that drive spleen plasticity remain to be determined. Social hierarchies have been previously shown to influence the immune cell repertoire and functions in rhesus macaques and humans [47,48]. Interestingly, proinflammatory and antibacterial phenotypes were shown in low-status individuals and antiviral phenotype in high-status individuals. Our findings suggest that social status might alter the immune system differently in NM-Rs compared to macaque and humans. It might be argued that stress hormones like glucocorticoid may be higher in lower-ranked individuals a factor known to be immunosuppressive. However, the available literature suggests that the levels of such hormones are not higher in lower-ranked individuals, but are actually boosted in individuals isolated from the colony [49].

We also compared the anatomical and molecular properties of the NM-R spleen to those of other rodents including mice, rats and two other social members of the Bathyergidae family to which NM-Rs belong [29]. Our analysis showed that among these rodents only the NM-R spleen display a dramatic decrease in white pulp/red pulp ratio [17,50] and a unique microarchitecture of the marginal zone (reduced marginal zone B cells and marginal zone macrophage populations) which might indicate that the clearance of blood-borne pathogens may be altered compared to other species. This phenotype might partially explain why NM-Rs readily succumb to herpes or coronavirus virus infections [15,16]. We also show that lymphopoiesis in NM-Rs is maintained since lymphocytes were found in peripheral blood, lymph nodes, mucosa-associated lymphoid tissues in the gut and the thymus, which all expressed CD3+ T cells. The thoracic thymus of NM-Rs was much smaller than those of mice at different ages. Cervical thymus could contribute to T cell maturation as observed in NM-Rs and other mammals [16,51,52]. It is well known that intrinsic and extrinsic factors such as cytokines and chemokines are involved in T and B cell migration and homing [31,53]. Our present data show that RNA levels of such factors (*S1pr1, S1pr3, Cxcl13, Cxcr5, Ccr7, Lta, Nkx2-3* and *Ctsb*) were reduced in NM-R spleens (electronic supplementary material, table S1). The mechanisms of tissue homing specificity observed in NM-R and the factors involved in this process remain to be determined.

We did not find reports describing viral or bacterial infections of NM-Rs in the wild [54–57], unlike their close relatives of the genus *Cryptomys* and *Bathyergus* that harbour *Bartonella* [58]. However, in captivity NM-Rs have been reported to be susceptible to coronavirus infection [15] and in our own laboratory we lost more than 55% of colony members (in colony 1: 20 out of 35 NM-Rs and in colony 2: 5 out of 8 NM-Rs) within a few months because of an unknown viral infection. Interestingly, in both laboratories after these mass die-off events almost all queens survived. The relative susceptibility of NM-Rs to viral infection may be due to a narrower immune cell spectrum available to eliminate pathogens with reductions in B cell lineages, dendritic cells, marginal zone macrophages and canonical NK [17] (and our present work). This is in contrast to observations of viral tolerance in some long-lived bat species [59]. However, we found an increase in gamma-Delta T cells (electronic supplementary material, figure S3E), a special T lymphocyte subset known to be at the border between evolutionary primitive innate system and the adaptive immune system and involved in the ‘first line of immune defence’ against viruses, bacteria and fungi [60,61]. The presence of more neutrophils and a LPS-responsive granulocyte population also support the idea of enhanced antibacterial defences in NM-Rs. Intriguingly, in injured NM-Rs despite increased numbers of immature neutrophils in peripheral blood indicating emergency myelopoiesis in response to injury [23], the animals did not recover, some even developed abscesses, suggesting increased vulnerability to secondary infection.

We also show that adult haematopoiesis takes place in NM-R spleen in addition to adult bone marrow haematopoiesis under normal physiological conditions, and regardless of spleen size. In rodents, extramedullary erythropoiesis is observed in response to hypoxia [33,62]. Thus, the active haematopoiesis in the spleen might reflect an adaptation of the NM-R to compensate for hypoxic environments. Surprisingly, despite the increase in splenic megakaryopoiesis a thrombocytopenia-like phenotype is observed in the peripheral blood of NM-Rs with low platelet counts and the presence of immature platelets. This could also be due to the hypoxic habitat of NM-Rs since in mice hypoxia induces thrombocytopaenia [63].

Interestingly, the NM-R immune system displays more similarities to humans than to that of other rodents with a larger myeloid compartment in peripheral blood and spleen, and insignificant splenic lymphopoiesis. Indeed, NM-R immature (stab-shaped neutrophils) and mature neutrophils found in bone marrow and in peripheral blood resemble those of humans [64]. Food, body size and physiology are factors known to influence spleen development [65]. We also found that thoracic thymus development was quite distinct in the NM-R compared to mouse. Hormonal and endocrine status can influence the development of the immune system [66] and it should be noted in this context that all NM-Rs used in this study were non-breeders and, therefore, reproductively suppressed [45]. We, like others have found low RNA levels of NK markers (*Ncr1, Nkg7* and *Gzma*). Furthermore, *Adgre1* expression (coding for F4/80), a known rapidly evolving gene in monocytic/macrophage lineages [67] and a marker of liver resident-macrophages and red pulp macrophages in mice, was almost absent in the spleen and liver of NM-Rs (electronic supplementary material, figure S7D and data not shown). This suggested that evolutionary pressure selected against the expression of such genes in the NM-R. Indeed, differences in phenotypic marker expression of immune cells between NM-R and mice should be treated with caution. Bone marrow macrophages of NM-R express the NK1.1 receptor of NK cells and are activated by NK1-1 antibodies *in vitro* [68]. We found low RNA levels of mouse classical red pulp macrophage markers (F4/80, *SpiC*, *Cd68*) (electronic supplementary material, figure S7D); however, GSEA and histological analyses showed that macrophages are present in the red pulp and they store iron (figures 4*i* and 6*e*; electronic supplementary material, table S4). Whether these macrophages resemble mouse red pulp macrophages remains to be determined. Interestingly, development and survival factors characteristic of the murine red pulp macrophages (*Irf8, Irf4, Bach1*) [42] were inversely expressed in NM-Rs compared to mice (electronic supplementary material, figure S7D and table S1). Unfortunately, we could not validate our data obtained on B cells, dendrite cells and macrophages due to a lack of specific reagents recognizing these immune cells in the NM-R.

Age-related changes of the immune system in humans and mice are thought to be caused by reduced thymus activity and chronic low-grade inflammation caused by increased activity of the innate immune system [69]. Interestingly, the composition of the NM-R spleen in healthy young animals is reminiscent of that of aged mice, including reduced abundance of marginal zone macrophages [70]. It remains to be seen whether the NM-R immune system is better equipped to prevent oncogenic events. Our observations of molecular and anatomical plasticity of the spleen in healthy higher-ranked animals raise the intriguing possibility that social success in this species may recruit the immune system to promote longevity.

## 4. Material and methods

### 4.1. Animals

Thirty-four healthy non-reproductive naked mole-rats (aged between 1.3 and 5 years; 18 males and 16 females) and 7 sick NM-Rs (aged between 1.7 and 7 years) were housed at the Max-Delbrück Center (MDC) in Berlin, Germany, in cages connected by tunnels, which were contained within a humidified incubator (50–60% humidity, 28–30°C), and heated cables ran under at least one cage per colony to allow for behavioural thermoregulation. Food (sweet potato, banana, apple, and carrot) was available ad libitum [71]. NM-Rs were sacrificed by decapitation.

Adult non-reproductive Natal mole-rats (*Cryptomys hottentotus natalensis*) and Highveld mole-rats (*Cryptomys hottentotus pretoriae*) were housed at the Department of Zoology and Entomology, University of Pretoria, South Africa in temperature-controlled rooms set at 25°C and a photoperiod of 12 h light–dark cycle. The humidity in the rooms was around 40–50%. mole-rats were fed on chopped vegetables and fruit daily and cleaned weekly with fresh wood shavings and paper towelling. All animals were humanly euthanized by decapitation under EC014-17.

Forty mice (aged between 4 weeks and 5 months, males and females) and 7 Sprague-Dawley rats (aged between 3 and 4.5 months, males and females) were fed *ad libitum* with standard diet and water on a 12 h light–dark cycle at 22°C ± 2°C under 55% ± 10% humidity. Mice and rats were housed in a pathogen-free facility at the MDC, Berlin, Germany. All procedures and animals experiments were conducted in compliance with protocols approved by the institutional Animal Care and Use Committee Landesamt für Gesundheit und Soziales Berlin (LAGeSo). Mice were sacrificed by cervical dislocation. Rats were sacrificed by decapitation with prior isoflurane anaesthesia. All efforts were made to minimize animal suffering.

### 4.2. Blood count, blood smear and bone marrow cell cytospin staining

Blood was collected after decapitation of NM-Rs directly into EDTA-containing tubes. Blood from mice was drawn via cardiac puncture and immediately transferred into EDTA-containing tubes. Blood cell counts were measured with an automated veterinary haematological counter Scil Vet abc (SCIL GmbH, Viernheim, Germany) or IDEXX ProCyte Dx haematology analyser (IDEXX, Germany) with software optimized for mouse blood parameters. May-Grünwald staining of blood smears was performed according to the manufacturer protocol (Sigma, Germany) and the cell type counts of the white blood cells were determined using a Leica DM 5000 B with a ×100 oil objective. At least 200 white blood cells were analysed per animal.

For performing cytospin and determining femur cellularity, bone marrow cells were flushed out from the femur, mechanically dissociated and counted using a TC20 automated cell counter (BioRad). For cytospin, 100 000 cells were centrifuged onto slides using a centrifuge slide stainer (Wescor) and stained manually with May–Grünwald staining.

### 4.3. Haematoxylin and eosin, iron staining, and immunostaining

Spleen, thymus, lymph nodes and small intestine Swiss rolls were rapidly collected, fixed overnight in 4% paraformaldehyde, embedded in paraffin, sectioned at 4 μm, and stained with haematoxylin & eosin histological stain according to the standard protocol. The histological detection of ferric iron in the spleen was performed using an iron staining kit (Abcam, cat. no. ab 150674).

For immunostaining, sections were deparaffinized and submitted to antigen retrieval (citrate buffer pH 6) using a microwave. After 2 washes with TBS-T (TBS with 0.05% Tween 20), sections were blocked with TBS-T + 5% goat serum for 30 min at room temperature and then incubated with rabbit primary antibodies overnight at 4°C. Primary antibodies were diluted in TBS-T + 1% goat serum. Sections were then washed three times with TBS-T, subsequently incubated with goat anti-rabbit-HRP (Jackson ImmunoResearch Labs, cat# 111-035-003, RRID:AB_2313567) for 1 h at room temperature. The rabbit primary antibodies CD3e (1 : 200, Abcam, cat. no. ab 5690, RRID:AB_305055) and MPO (1 : 500, Dako/Agilent, cat. no. A0398, RRID:AB_2335676) were used. Dako-EnVision + System-HRP (Dako, cat. no. K4002) was used for immunodetection. Haematoxylin counter staining was performed before mounting. All images were acquired using a Leica DM 5000 B. To quantify the number of splenic megakaryocytes, four randomly chosen fields in red pulp were photographed at 40× magnification for each animal and analysed using Item 5 software program (v. 5).

### 4.4. RNA preparation and RNA sequencing

Total RNA was isolated from three biological replicates per species and per group using RNeasy extraction kit (Promega). RNA-seq libraries were prepared using the Truseq Stranded total RNA kit (Illumina) and sequenced on the Illumina NovaSeq 6000 platform according to the manufacturer's instruction at Macrogen (Macrogen, Korea). Reads were aligned to mm9 and hetGla2/hetGla Female_1.0, respectively, using STAR aligner v. 2.5.3a. The aligned reads were then transformed to raw count tables using htseq-count version 0.10.0. The raw and normalized data are deposited at Gene Expression Omnibus (GEO, accession number GSE179350).

### 4.5. Transcriptomic analysis

Pre-processed RNA-seq data were imported in R (v. 3.5.1) for downstream analysis. NM-R genes were annotated to *Mus musculus* homologue-associated gene names using the biomaRt package (v. 2.38.0) to allow merging of the mouse and NM-R data sets. Genes were pre-filtered to remove those transcripts not corresponding to gene symbols or not reaching read sums higher than 10 across all samples. The DESeq2 package (v. 1.22.2) served for normalization and differential expression analysis. Differentially expressed genes were called using a threshold of an adjusted *p*-value (*p*_adj_) < 0.05 after multiple-testing correction (Benjamini–Hochberg). For global expression analysis principal component analysis was done using pcaExplorer (v. 2.8.1) based on the top 3000 variable genes and a global distance matrix was generated using the Euclidean distance. Expression of gene sets was visualized using the pheatmap package (v. 1.0.12), gene wise scaling and Pearson correlation as distance measure for hierarchical clustering where applicable. To perform gene set enrichment analysis the fgsea package (v. 1.8) was used and pre-built, established gene sets were applied (available on request) or custom gene sets were generated from published data derived from NM-R transcriptomes [17,72]. To assess the cellular composition within the spleens based on the bulk transcripome data, CIBERSORT analysis was performed using the web interface as described by Newman *et al.* [25]. For this, mouse immune gene expression signatures were used as presented by Chen *et al.* [26].

### 4.6. Immunoblotting

Tissues were lysed with 8 M urea and protein analysed by SDS/PAGE/protein blotting using rabbit antibody against CD3e (Abcam, cat. no. ab 5690, RRID:AB_305055), rabbit antibody against anti-human MPO (Dako/Agilent, cat. no. A0398, RRID:AB_2335676), mouse β-actin (Sigma-Aldrich, cat. no. A1978, RRID:AB_476692), horseradish peroxidase-conjugated secondary antibodies (Jackson ImmunoResearch Labs, cat# 111-035-003, RRID:AB_2313567) and chemiluminescence detection (Thermo Fischer).

### 4.7. Hierarchy assessment and ranking index

Methods were as described in [13] and modified from [45]. In brief, two NM-Rs were allowed to approach each other head on in an artificial plastic tunnel. During these interactions the more dominant individual will reliably climb over the subordinate individual. Using a single-elimination strategy, with a minimum of three trials for each pseudo-randomly selected pairing of NM-Rs from a single colony, a ranking index (RI) was calculated for each colony. RI = (number of wins) divided by (the total number of behavioural trials). RI values were normalized to the maximum value for each colony and the following rankings were assigned based on RI: rank 1, RI > 0.8, rank 2, RI > 0.6, rank 3, RI > 0.4, rank 4, RI > 0.2, rank 5, RI < 0.2. The queen was assigned a rank of 1.

### 4.8. Statistical analysis

All data are expressed as mean ± s.e.m. Data were first tested for normal distribution. For CIBERSORT analysis variation is reported as ± standard deviation. Statistical tests performed can be found in the Figure legends. Statistical analyses were carried out using Prism 8 (GraphPad Prism, RRID:SCR_002798) unless otherwise stated. *p* value < 0.05 was considered to be statistically significant.

## Data Availability

The raw and normalized data are deposited at Gene Expression Omnibus (GEO, accession no. GSE179350). The data are provided in electronic supplementary material [73].
